# Tumor Necrosis Factor-α Regulates Distinct Molecular Pathways and Gene Networks in Cultured Skeletal Muscle Cells

**DOI:** 10.1371/journal.pone.0013262

**Published:** 2010-10-12

**Authors:** Shephali Bhatnagar, Siva K. Panguluri, Sanjay K. Gupta, Saurabh Dahiya, Robert F. Lundy, Ashok Kumar

**Affiliations:** Department of Anatomical Sciences and Neurobiology, University of Louisville School of Medicine, Louisville, Kentucky, United States of America; Johns Hopkins School of Medicine, United States of America

## Abstract

**Background:**

Skeletal muscle wasting is a debilitating consequence of large number of disease states and conditions. Tumor necrosis factor-α (TNF-α) is one of the most important muscle-wasting cytokine, elevated levels of which cause significant muscular abnormalities. However, the underpinning molecular mechanisms by which TNF-α causes skeletal muscle wasting are less well-understood.

**Methodology/Principal Findings:**

We have used microarray, quantitative real-time PCR (QRT-PCR), Western blot, and bioinformatics tools to study the effects of TNF-α on various molecular pathways and gene networks in C2C12 cells (a mouse myoblastic cell line). Microarray analyses of C2C12 myotubes treated with TNF-α (10 ng/ml) for 18h showed differential expression of a number of genes involved in distinct molecular pathways. The genes involved in nuclear factor-kappa B (NF-kappaB) signaling, 26s proteasome pathway, Notch1 signaling, and chemokine networks are the most important ones affected by TNF-α. The expression of some of the genes in microarray dataset showed good correlation in independent QRT-PCR and Western blot assays. Analysis of TNF-treated myotubes showed that TNF-α augments the activity of both canonical and alternative NF-κB signaling pathways in myotubes. Bioinformatics analyses of microarray dataset revealed that TNF-α affects the activity of several important pathways including those involved in oxidative stress, hepatic fibrosis, mitochondrial dysfunction, cholesterol biosynthesis, and TGF-β signaling. Furthermore, TNF-α was found to affect the gene networks related to drug metabolism, cell cycle, cancer, neurological disease, organismal injury, and abnormalities in myotubes.

**Conclusions:**

TNF-α regulates the expression of multiple genes involved in various toxic pathways which may be responsible for TNF-induced muscle loss in catabolic conditions. Our study suggests that TNF-α activates both canonical and alternative NF-κB signaling pathways in a time-dependent manner in skeletal muscle cells. The study provides novel insight into the mechanisms of action of TNF-α in skeletal muscle cells.

## Introduction

Skeletal muscle atrophy or wasting is a common phenomenon in a large number of systemic diseases including sepsis, diabetes, chronic obstructive pulmonary disease, heart failure, and cancer [1], [2], [3], [4]. Accumulating evidence suggests that inflammatory cytokines especially TNF-α play a major role in the development of muscular abnormalities resulting in loss of skeletal muscle mass and function [5]. Increased levels of TNF-α have been observed under conditions that lead to skeletal muscle atrophy such as chronic heart failure, cancer, AIDS, and cachexia induced by bacteria [6]. TNF-α transduces its biological activities by binding to two 55- and 75-kDa receptors [7]. Trimeric occupation of TNF receptors by the ligand results in the recruitment of receptor-specific proteins leading to the activation of a cascade of protein kinases such as IκB kinase (IKK), transforming growth factor-β activated kinase 1 (TAK1), mitogen-activated protein kinases (MAPKs), and Akt and several downstream transcription factors [7], [8], [9].

Nuclear factor-kappa B (NF-κB) is a major proinflammatory transcription factor that regulates the expression of a plethora of genes especially those involved in inflammatory and immune responses [10], [11]. Depending on the type of stimuli, the activation of NF-κB can occur via either canonical or alternative pathway [10]. The canonical NF-κB signaling pathway involves the upstream activation of inhibitors of κB (IκB) kinase-β (IKKβ) and subsequent phosphorylation and degradation of IκB proteins. On the other hand, activation of the alternative NF-κB pathway requires the upstream activation of NF-κB-inducing kinase (NIK) and IKKα and the proteolytic processing of p100 subunit into p52 [10], [12]. Several recent studies have provided strong evidence that constitutive activation of NF-κB leads to skeletal muscle wasting and its inhibition prevents the loss of skeletal muscle mass in response to various catabolic stimuli including TNF-α [10], [13], [14], [15], [16], [17]. Li et al [17] showed that TNF-α-induced activation of NF-κB is responsible for the up-regulation of ubiquitin-conjugating E2 enzyme UbcH2 resulting in increased activity of ubiquitin-proteasome system and degradation of myofibril proteins. Furthermore, the inhibitory effect of TNF-α on myogenesis is mediated through the activation of NF-κB which downregulates the levels of myogenic regulatory factor MyoD in myoblasts through distinct mechanisms [18], [19], [20].

It is also noteworthy that the catabolic action of TNF-α in skeletal muscle may require the presence of other proinflammatory cytokines, such as TNF-related weak-inducer of apoptosis (TWEAK), interleukin-1β (IL-1β), interleukin-6 (IL-6), and interferon γ (IFN-γ) [16], [21], [22], [23]. A combination of TNF-α and IFN-γ has been reported to cause a strong down-regulation of muscle specific gene products including MyoD in cultured muscle cells [18]. However, it is not yet clear whether TNF-α augments the expression of other inflammatory cytokines or they are expressed by independent mechanisms in skeletal muscle cells.

Recent investigations involving genome-wide gene expression profiling in skeletal muscle has helped in identifying several novel genes which mediate the loss of skeletal muscle mass in different muscle-wasting conditions [24], [25], [26], [27], [28]. However, the effects of TNF-α on the global gene expression and intracellular pathways that it affects in skeletal muscle remain poorly understood. To attain a better molecular insight into the mechanisms of action of TNF-α in skeletal muscle, we focused the present investigation on identification of TNF-regulated gene expression, gene networks, and molecular pathways in skeletal muscle. Microarray analyses of control and TNF-treated myotubes revealed that TNF-α regulates the expression of several genes and pathways which may be related to its catabolic action in skeletal muscle. Furthermore, our study provides the initial evidence that TNF-α activates both canonical and alternative NF-κB signaling pathways in skeletal muscle cells.

## Results

We have used microarray approach to identify the set of genes which TNF-α regulates in cultured C2C12 myotubes. To detect the expression of both early and late responsive genes, we have performed mRNA profiling after 18h of TNF-α treatment. Analysis using MTT [3-(4,5-dimethyl thiazol-2-yl)-2,5-diphenyl tetrazolium bromide] dye showed no significant difference in cell viability between control and TNF-treated C2C12 myotube cultures after 18h (not depicted). Raw and normalized data of this microarray experiment has been submitted to ArrayExpress database (http://www.ebi.ac.uk/microarray-as/ae/) with accession number E-MEXP-2592.

### Identification of differentially expressed genes in TNF-treated C2C12 myotubes by microarray technique

**Figure 1:**
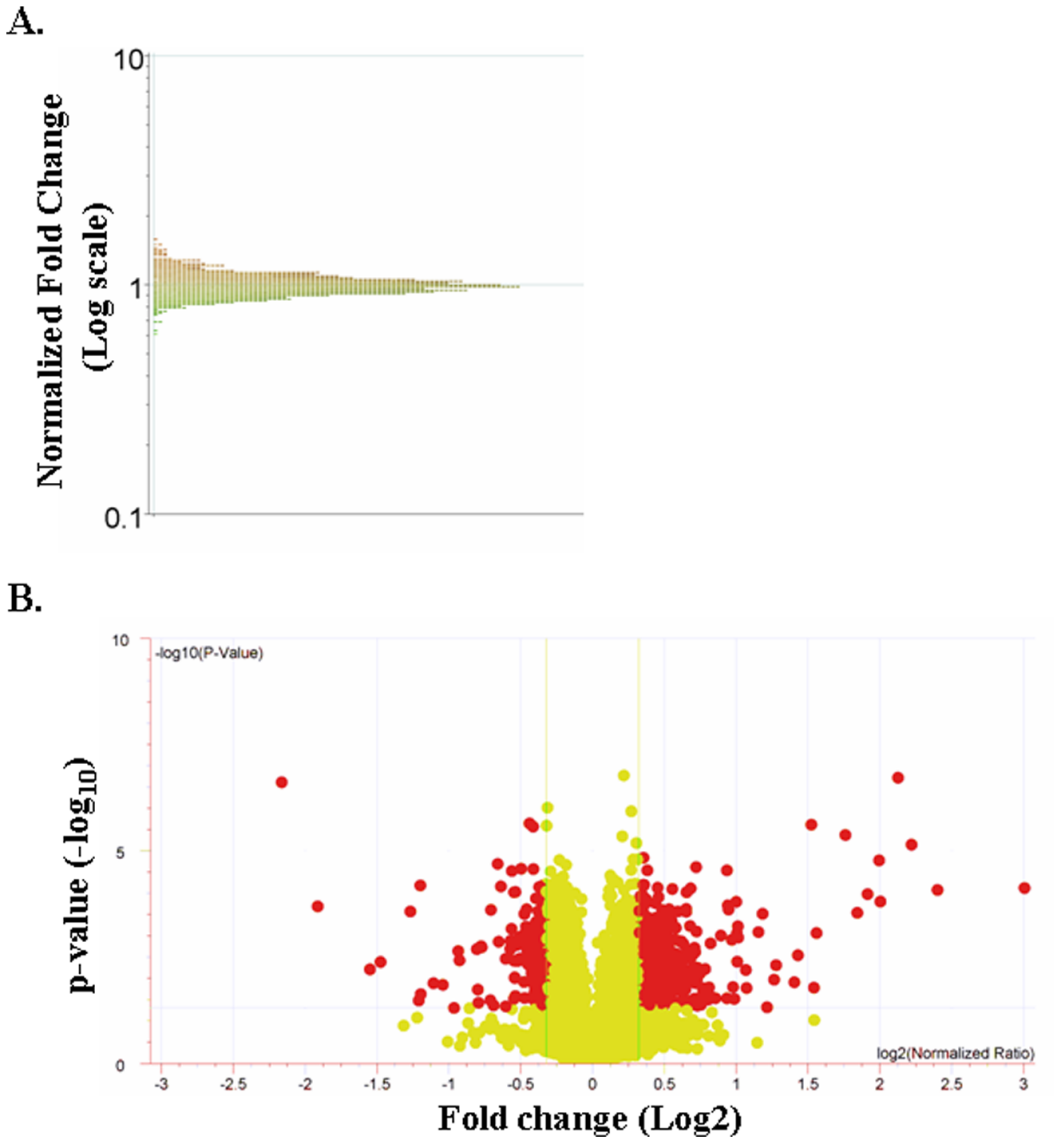
Gene distribution and volcano plots of differentially expressed genes in TNF-treated C2C12 myotubes. **A**) Distribution curve of differentially expressed genes in response to TNF treatment detected by cDNA microarray analysis. The normalized fold changes were plotted on y-axis on logarithmic scale. **B**) Volcano plots of differentially expressed genes in response to TNF treatment. Here −log10 p-values were plotted on y-axis and log2 values of normalized ratios were plotted on x-axis.

C2C12 myotubes were treated with TNF-α (10ng/ml) for 18h and the mRNA levels of different genes were monitored by cDNA microarray technique. The microarray gene expression profile appeared normally distributed for TNF-treated samples (**Figure 1A**) indicating that our analyses of differentially expressed genes is not biased due to skewed distribution of certain genes. Out of approximately 25,000 genes present on our microarray chips, TNF-α significantly (p<0.05) affected the expression of a total of 5,939 genes, out of which 3,349 genes were down regulated and 2,590 genes were up regulated. We have also filtered the genes with the fold change ≥1.2 or ≤1.2, which yielded 1,822 differentially regulated genes. In particular, 723 genes were significantly down regulated whereas 1,099 genes were significantly up regulated by TNF treatment. The volcano plot of differentially expressed genes with these cut-off p-values and fold changes is presented in the **Figure 1B**. Further analysis of differentially regulated genes showed that about 51 genes were differentially regulated by TNF with p-values ≤0.0001 (28 up-regulated and 23 down-regulated with fold values ≥1.2), 181 genes with p-value ≤0.001 (91 up-regulated and 90 down-regulated), 751 genes with p-values ≤0.01 (406 up-regulated and 345 down-regulated), and 839 genes with p-values ≤0.05, of which 574 genes were up-regulated and 265 genes were down-regulated (**Table 1**). The functional annotations of important genes differentially expressed in TNF-treated myotubes are presented in the **Table S1**. Several genes such as transforming growth factor beta 3 or TGF-β3 (−1.28 fold), tissue inhibitor of metalloproteinase 1 or TIMP1 (1.6 fold), nuclear factor of kappa light chain gene enhancer in B-cells inhibitor, alpha or IκBα (3.98 fold), and chemokine (C-X-C motif) ligand 5 or Cxcl5 (8 fold) are with p-values ≤0.0001. Genes such as inhibitor of DNA binding 3 or ID3 (−1.63 fold), protein kinase inhibitor, alpha or PIKα (−1.38 fold), cyclin-dependent kinase inhibitor 1C or Cdkn1c (−1.3 fold), forkhead box d2 or FoxD2 (−1.27 fold), nuclear factor related to kappa B binding protein or NFRκB (−1.28 fold), B-cell leukemia/lymphoma-6 or BCL6 (−1.24 fold), jagged 2 or Jag2 (−1.22 fold), vascular endothelial growth factor c or VEGFc (1.28 fold), insulin-like growth factor binding protein 7 or IGFbp7 (1.6 fold), tumor necrosis factor-alpha induced protein-3 or TNFαIP3 (1.6 fold), nuclear factor of kappa light chain gene enhancer in B-cells 1, p105 or NFκB1 (1.6 fold), colony stimulating factor or CSF1 (2-fold), chemokine (C-C motif) ligand 5 or Ccl5 (3.6 fold), and chemokine (C-C motif) ligand 2 or Ccl2 (3.8 fold) were with p-values ≤0.001. Among the genes with p-values ≤0.01, notch homolog1 or Notch1 (−1.6 fold), procollagen, type IV or Col4a2 (−1.4 fold), early growth response 1 or EGR1 (−1.3 fold), adenylate cyclase 9 or Adcy9 (−1.3 fold), insulin-like growth factor binding protein-6 or IGFbp6 (−1.25 fold), tumor necrosis factor receptor superfamily, member23 or Tnfrsf23 (−1.25 fold), forkhead box O6 or FoxO6 (−1.23 fold), TGF-β induced early growth response 3 or Tieg3 (−1.2 fold), signal transducer and activator of transcription 5a or STAT5a (1.2 fold), tachykinin receptor 3 or Tacr3 (91.2 fold), forkhead boxD3 or FoxD3 (1.23 fold), breast cancer 1 or BRCA1 (1.27 fold), a disintegrin and metalloprotease domain 29 or Adam29 (1.3 fold), cAMP response element binding protein 3 or CREB3 (1.3 fold), myocyte enhancer factor 2B or Mef2B (1.32 fold), Maf oncogene or v-maf (1.36 fold), chemokine (C-X-C motif) receptor 3 or Cxcr3 (1.38 fold), nuclear factor of kappa light polypeptide gene enhancer in B-cells 2, p49/100 or NFκB2 (1.4 fold), matrix metalloproteinase-9 or MMP-9 (1.5 fold), interleukin-6 or IL-6 (1.66 fold), and TAF2 RNA polymerase II, TATA box binding protein (TAB)-associated factor, 150kDa (2.7 fold) are important genes regulating many pathways. Similarly, myogenic differentiation 1 or MyoD1 (−1.3 fold), mitogen activated protein kinase 4 or MAPK4 (−1.3 fold), myocyte enhancer factor 2c or Mef2c (−1.27 fold), CD27 binding protein or Siva (−1.24 fold), superoxide dismutase 1 or SOD1 (−1.23 fold), Jun oncogene (−1.2 fold), histone deacetylase 10 or HDAC10 (−1.2 fold), and vascular cell adhesion molecule 1 or Vcam1 (2.91 fold) are very important genes differentially regulated by TNF-α with p-values ≤0.05.

**Table 1:** Number of differentially expressed genes with different p-values and ≥1.2 fold in TNF-α treated C2C12 myotubes.

p-value	Up regulated genes	Down regulated genes	Total genes
≤0.0001	28	23	51
>0.0001 but ≤0.001	91	90	181
>0.001 but ≤0.01	406	345	751
>0.01 to 0.05	574	265	839

**Figure 2:**
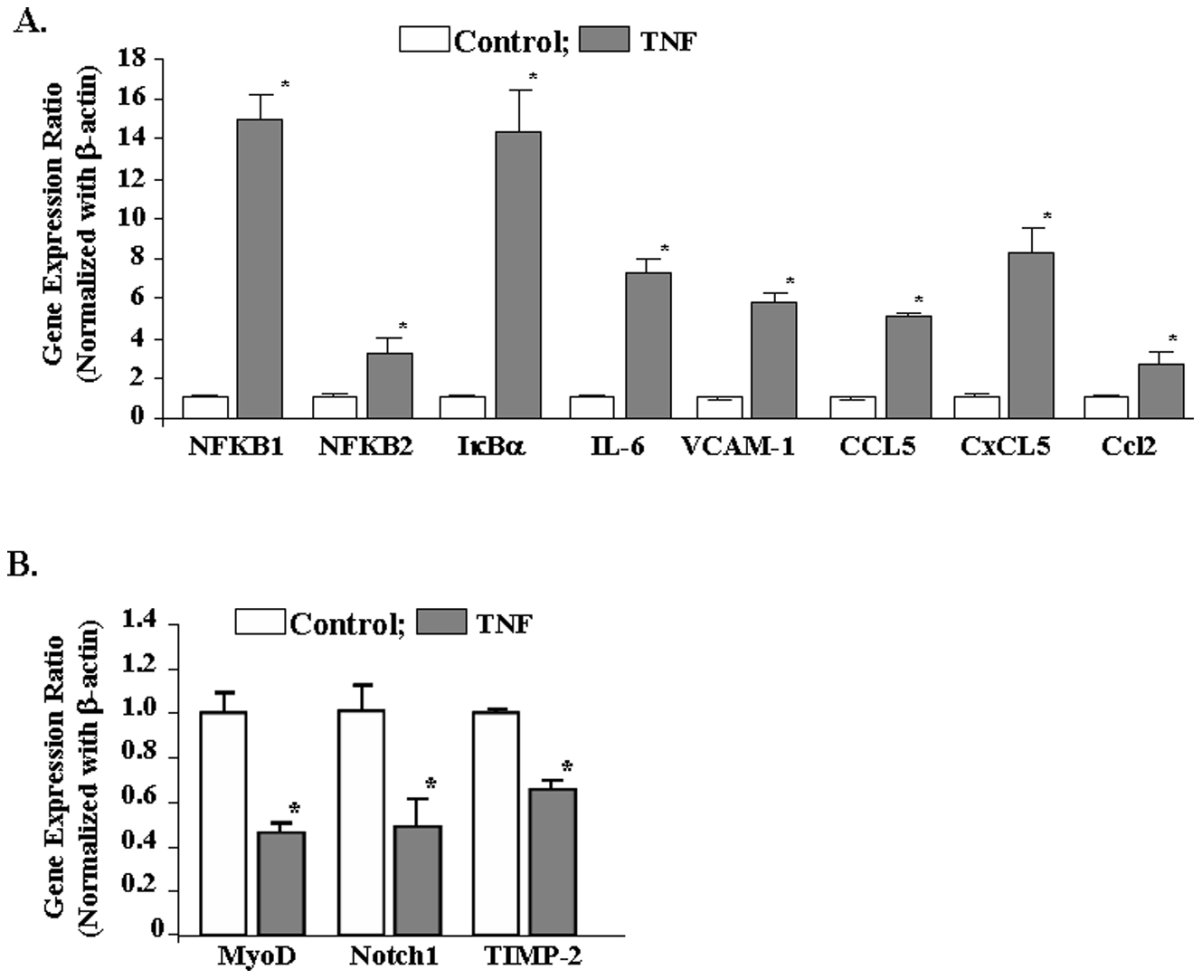
Validation of genes differentially regulated by TNF-α by QRT-PCR. C2C12 myotubes were treated with 10 ng/ml of TNF for 18h followed by isolation of total RNA and QRT-PCR. Untreated myotubes under similar conditions were taken as control. The relative expression values (normalized with β-actin) from the QRT-PCR analysis were plotted for each gene are mean ± SD (n = 3). ‘*’ represents the statistical significance (p-value ≤0.01). **A**). Data presented here show that mRNA levels of NFKB1, NFKB2, IκBα, IL-6, VCAM1, Ccl5, Cxcl5, and Ccl2 were significantly increased in TNF-treated C2C12 cells. **B**). Data presented here show that mRNA levels of MyoD, Notch1, and Timp-2 were significantly reduced in TNF-treated C2C12 myotubes.

We next sought to determine whether the expression levels of some of the genes which showed significant up- or down-regulation in microarray experiment can be recapitulated in independent quantitative real time-PCR (QRT-PCR) assays. QRT-PCR assays were performed for the genes which showed high fold change and/or have a direct or indirect relation with skeletal muscle wasting. As shown in **Figure 2A**, the expression of Nfkbia (also known as IκBα), Nfkb1, Nfkb2, IL-6, Vcam1, Ccl5, Cxcl5, and Ccl2 was found to be significantly increased in TNF-treated samples in QRT-PCR assays. Similarly, the reduced expression of Notch1, TIMP2, and MyoD in TNF-treated samples was confirmed by independent QRT-PCR assays (**Figure 2B**) suggesting a direct correlation between microarray and QRT-PCR analysis for almost all the genes tested.

**Figure 3:**
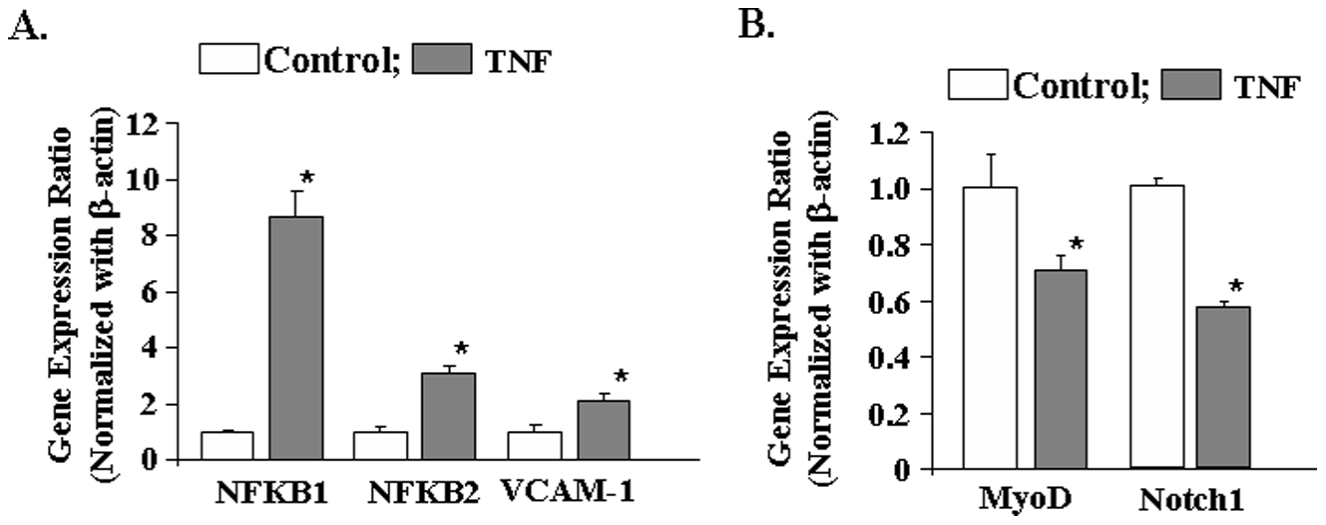
Effects of TNF-α on gene expression in C2C12 myoblasts. C2C12 myoblasts incubated in growth medium were treated with 10 ng/ml TNF-α for 18h followed by isolation of total RNA and performing QRT-PCR assay. **A**). Data presented here demonstrate that TNF-α significantly augments the expression of NFKB1, NFKB2, and VCAM-1 in C2C12 myoblasts. **B**). Data presented here demonstrate TNF-α significantly reduces the mRNA levels of MyoD and Notch1 in C2C12 myoblasts. *p<0.05, values significantly different from control myoblasts incubated without TNF-α.

C2C12 myoblasts differentiate into myotubes in low serum conditions. However, the differentiation of C2C12 myoblasts into myotubes is never complete [29]. C2C12 cultures incubated in differentiation medium still contain a significant number of undifferentiated myoblasts [29]. We investigated whether the observed changes in gene expression in response to TNF-α occur in undifferentiated myoblasts or myotubes or both in C2C12 cultures. To answer this question, we first studied whether TNF-α can affect the gene expression in undifferentiated C2C12 myoblasts incubated in growth medium. Treatment with TNF-α significantly increased the mRNA levels of NFKB1, NFKB2, and VCAM-1 (**Figure 3A**) and reduced the levels of MyoD and Notch1 (**Figure 3B**) in C2C12 myoblasts.

**Figure 4:**
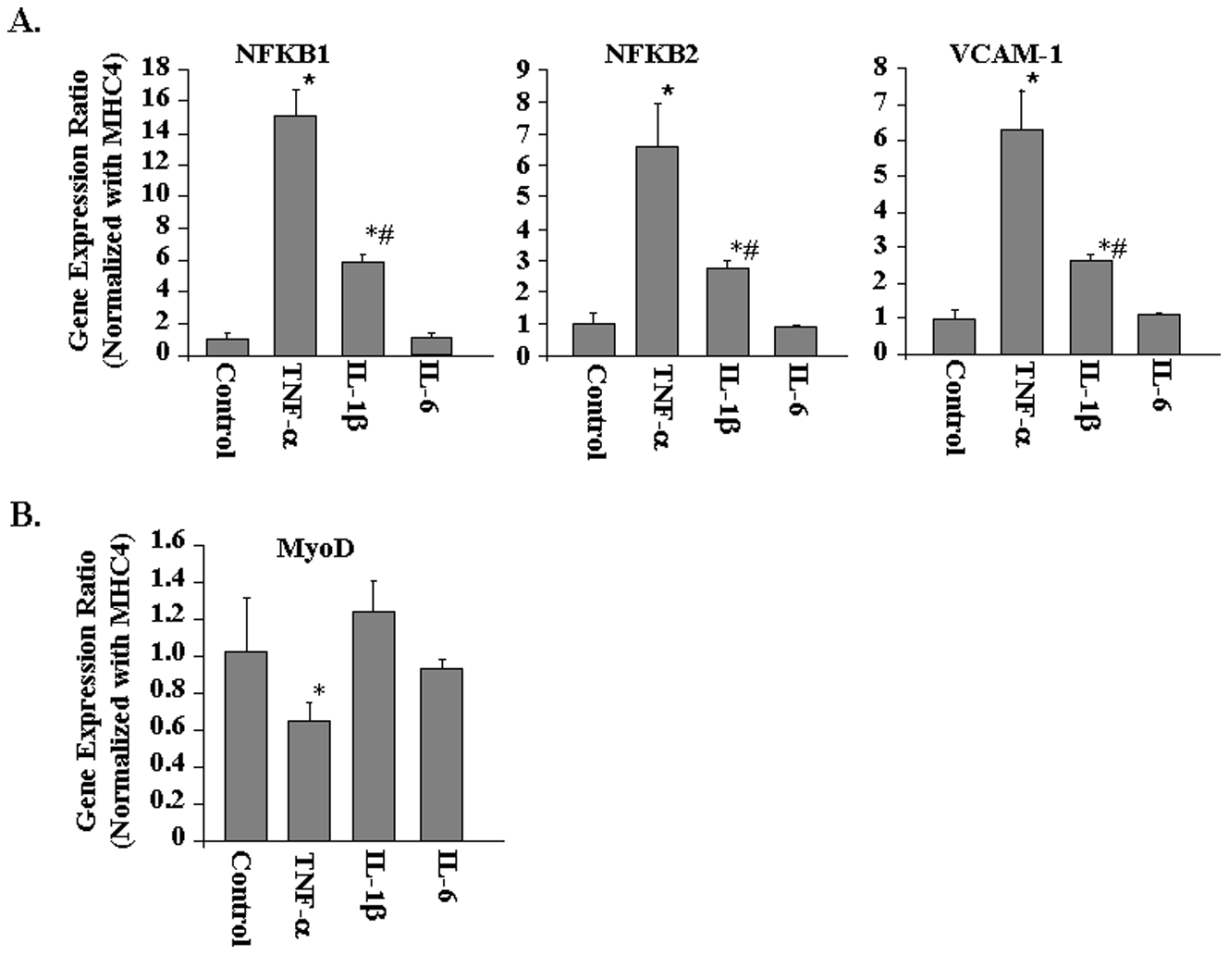
Effects of TNF-α, IL-1β, and IL-6 on gene expression in C2C12 myotubes. C2C12 myotubes were treated with 10 ng/ml TNF-α, IL-1β, or IL-6 for 18h followed by total RNA isolation and performing QRT-PCR using myosin heavy chain 4 (MYH4) as the normalizing gene. **A**). The fold increase in mRNA levels of NFKB1, NFKB2, and VCAM-1 compared to untreated controls is presented here. **B**). Fold change in mRNA levels of MyoD compared to untreated controls. *p<0.01, values significantly different from untreated myotubes. ^#^p<0.01, values significantly different from TNF-treated myotubes for respective gene.

To evaluate whether TNF-α affects gene expression in myotubes, we performed QRT-PCR assays for a few select genes using myosin heavy chain 4 (MHC4, expressed only in differentiated muscles) as the normalizing gene. The mRNA levels of NFKB1, NFKB2, and VCAM-1 were significantly higher (**Figure 4A**) and that of MyoD were significantly lower (**Figure 4B**) in TNF-treated cultures compared to untreated cultures. Taken together, these results indicate that TNF-α modulates gene expression in both myotubes and myoblasts in a similar fashion.

In addition to TNF-α, several other proinflammatory cytokines such as IL-1β and IL-6 have been postulated to be the mediators of muscle-wasting in various chronic diseases [6]. We investigated whether IL-1β and IL-6 can also modulate gene expression in C2C12 cultures similar to TNF-α. Interestingly, we observed that IL-1β but not IL-6 significantly augmented the expression of NFKB1, NFKB2, and VCAM-1 in C2C12 cultures. However, the fold increase in their mRNA levels in response to IL-1β was significantly lower compared to TNF-α (**Figure 4A**). Moreover, we found that while TNF-α significantly reduced mRNA level of MyoD in C2C12 myotubes, neither IL-1β nor IL-6 had any significant effect on the expression of MyoD (**Figure 4B**). The data suggest that TNF-α may be the most potent stimulus whereas other cytokines may also contribute to muscle wastage to some extent.

**Figure 5:**
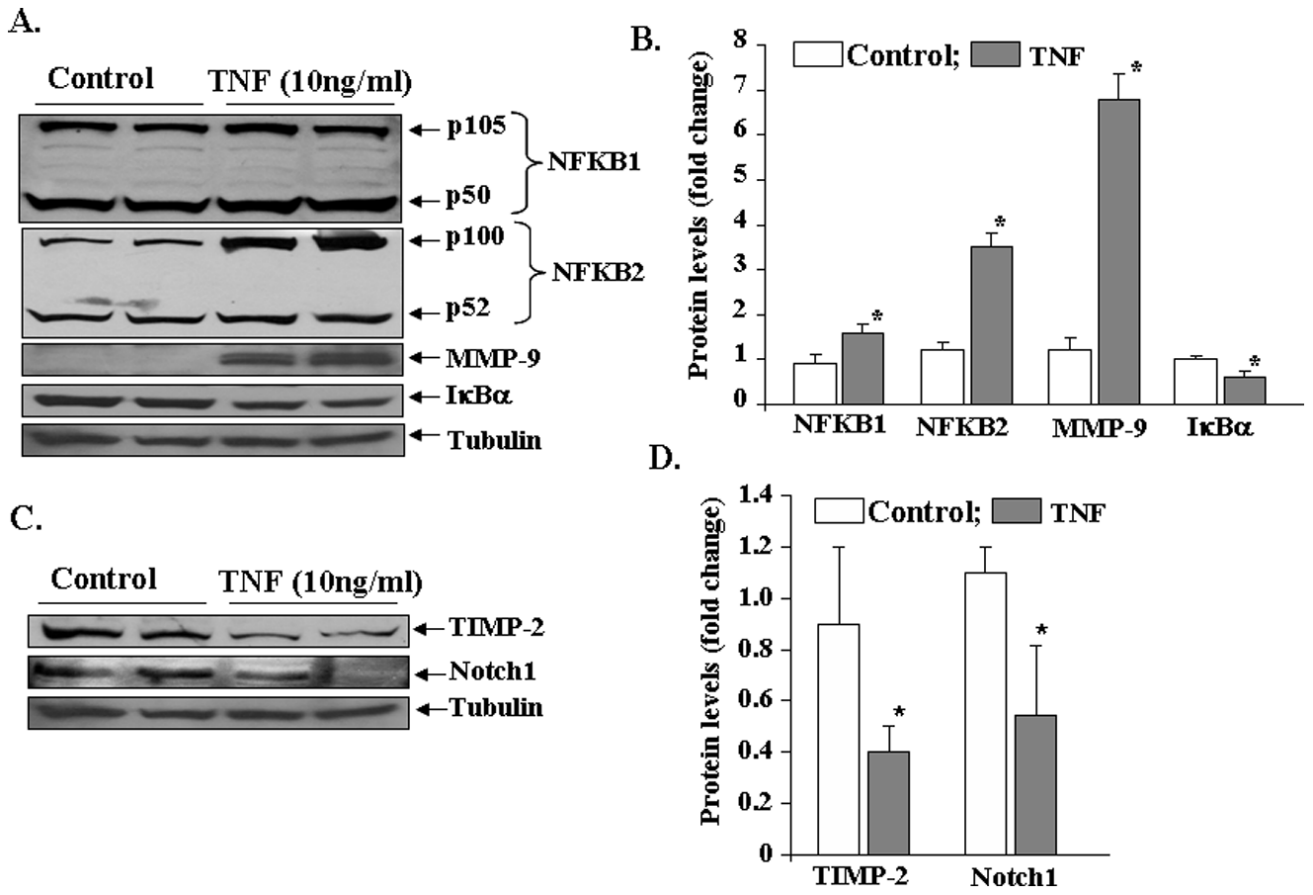
Western blot analyses of genes differentially expressed by TNF-α in myotubes. C2C12 myotubes were treated with 10 ng/ml of TNF for 18h followed by isolation of total protein and Western blotting. Equal amounts of proteins were loaded on 10% SDS-PAGE gel. **A**). Representative immunoblots presented here show that TNF-α increases the protein levels of NFKB1, NFKB2, MMP-9, and IκBα in myotubes. **B**). Densitometry analyses of bands from NFKB1, NFKB2, MMP-9, and IκBα immunoblots. **C**). Protein levels of Timp-2 and Notch1 were found to be reduced upon treatment with TNF-α. D). Densitometry analyses of bands from TIMP-2 and Notch1 immunoblots. *p<0.05, values significantly different from corresponding control myoblasts incubated without TNF-α (n = 4 in each group).

To further confirm our microarray findings, we also performed Western blots for a few select proteins affected by TNF-α. Consistent with their mRNA levels, the protein levels of NFKB1, NFKB2, and MMP-9 were significantly increased in TNF-treated myotubes compared to untreated myotubes (**Figures 5A and 5B**). It was interesting to note that while TNF-α increased the mRNA levels of NFKB1 by ∼15 fold, the increase in NFKB1 protein level was only ∼1.6 fold (**Figures 5A and 5B**). Although the exact reasons remain unknown, it is possible that in addition to increasing the expression, TNF-α also increases the turnover of NFKB1 protein. Furthermore, there is also a possibility that NFKB1 mRNA is subjected to post-transcriptional modifications (including those involving micro RNAs) which may limit its translation into protein. In contrast to mRNA levels, the protein levels of NF-κB inhibitor IκBα was found to be significantly reduced (**Figures 5A and 5B**). The reduced levels of IκBα in TNF -treated myotubes could be attributed to its enhanced degradation in response to NF-κB activation stimuli [10], [12]. In agreement with microarray data, we found reduced protein levels of Notch1 and TIMP-2 in TNF-treated myotubes (**Figures 5C, and 5D**).

### TNF-α causes activation of both canonical and alternative NF-κB signaling pathways in myotubes

**Figure 6:**
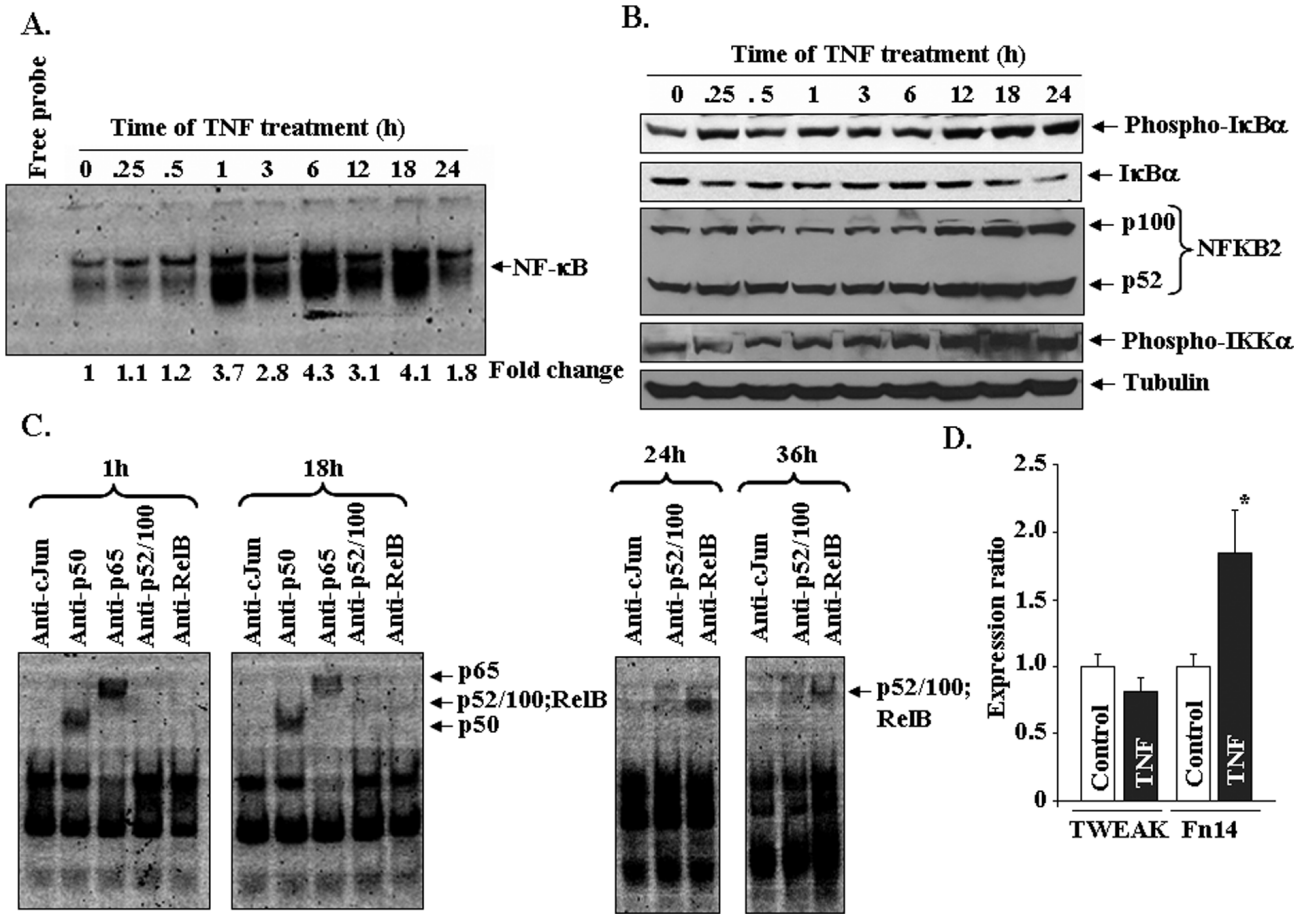
TNF-α activates canonical and alternative NF-κB pathways in C2C12 myotubes. **A**) C2C12 myotubes were treated with 10 ng/ml of TNF for indicated time periods and the activation of NF-κB was measured by EMSA. The representative EMSA gel presented here shows that there is a significant increase in NF-κB activity in myotubes upon treatment with TNF-α. **B**) C2C12 myotubes were treated with 10 ng/ml of TNF-α for indicated time followed by performing Western blots using anti-IκBα, anti-p100/52, and anti-phospho-IKKα. **C**). Analysis of NF-κB/DNA complex by super shift assays in myotubes treated with TNF-α for different lengths of time. **D**). The relative expression of TWEAK and Fn14 in control and TNF-treated myotubes determined by QRT-PCR. *p<0.05, values significantly different from untreated myotubes.

TNF-α is a well-known activator of canonical NF-κB signaling pathway which involves the upstream activation of IκB kinase-β (IKKβ) and subsequent phosphorylation and degradation of IκBα protein [7]. Although it has been reported that TNF-α activates NF-κB in skeletal muscle cells [7], [10], [17], [30], there has been no report whether TNF-α can augment the activation of alternative NF-κB signaling pathway which involves the activation of IKKα and proteolytic processing of p100 subunit into p52. Surprisingly, our microarray and subsequent QRT-PCR and Western blot revealed increased expression of both NFKB1 (e.g. p105/p50) and NFKB2 (e.g. p100/p52) in TNF-treated myotubes (**Figures 2** **,** **4** **, and** **5**). These results prompted us to investigate whether TNF-α activates both canonical and alternative NF-κB pathways and the time points at which these two pathways are up-regulated after treatment with TNF-α. C2C12 myotubes were treated with TNF-α for different time periods ranging from 0–24h and the activation of NF-κB was measured by electrophoretic mobility shift assays (EMSA). As shown in **Figure 6A**, treatment with TNF-α led to sustained activation of NF-κB in C2C12 myotubes though it peaked at 1h, 6h and 18h. These results are consistent with a previously published report also demonstrating biphasic activation of NF-κB in C2C12 myotubes upon TNF-α-treatment [31]. To investigate whether TNF-α-induced activation involves canonical, alternative, or both pathways, we performed Western blot using TNF-α-treated myotubes. As shown in **Figure 6B**, treatment with TNF-α increased the phosphorylation and reduced the levels of IκBα protein. IκBα protein levels remained lower compared to untreated cultures indicating that TNF-α stimulates the canonical NF-κB signaling pathway in myotubes. Interestingly, we found that TNF-α did not affect the expression or proteolytic processing of p100 subunit into p52 up to 6h. However, after 6h, a significant increase was noticed in protein levels of both p100 and p52 suggesting the activation of alternative NF-κB pathway. IKKα is the kinase which phosphorylates p100 protein leading to its proteolytic processing into p52 subunit [10]. To further investigate whether the activation of IKKα is increased in response to TNF-α treatment, we performed Western blot using antibody which recognizes the phosphorylated (activated) IKKα protein. As shown in **Figure 6B**, TNF-α-induced the activation of IKKα in a time-dependent manner. Consistent with proteolytic degradation of p100 protein, the activation of IKKα was noticeable at 12h and later time points (**Figure 6B**).

To further confirm that TNF-α activates both canonical and alternative NF-κB signaling pathways, we also performed super-shift assays using nuclear extracts from TNF-treated myotubes. As shown in **Figure 6C**, incubation of nuclear-extracts from 1h and 18h TNF-treated myotubes with anti-p50 and anti-p65 shifted the NF-κB/DNA complex to higher molecular weight indicating that activated NF-κB complex contains p50 and p65 (the components of canonical NF-κB pathway) proteins. Although a slight shift was observed with antibodies against RelB and p52 at 18h, a remarkable increase in shifted bands was evident with both the antibodies at 24h and 36h after treatment with TNF-α. These results suggest that TNF-α initially activates canonical NF-κB pathway followed by the activation of alternative pathway.

We have recently reported that TWEAK, a member of TNF super family, is a potent activator of alternative NF-κB signaling pathway in skeletal muscle cells [32]. By performing QRT-PCR, we investigated whether TNF-α augments the expression of TWEAK or its receptor Fn14 in C2C12 myotubes. Our results showed that the mRNA levels of TWEAK were comparable between control and TNF-treated myotubes (**Figure 6D**). Furthermore, we could not detect TWEAK protein in culture supernatants of control or TNF-treated myotubes by ELISA. However, QRT-PCR assays showed a moderate increase (∼1.7 fold) in the mRNA levels of Fn14 in TNF-treated myotubes (**Figure 6D**).

**Figure 7:**
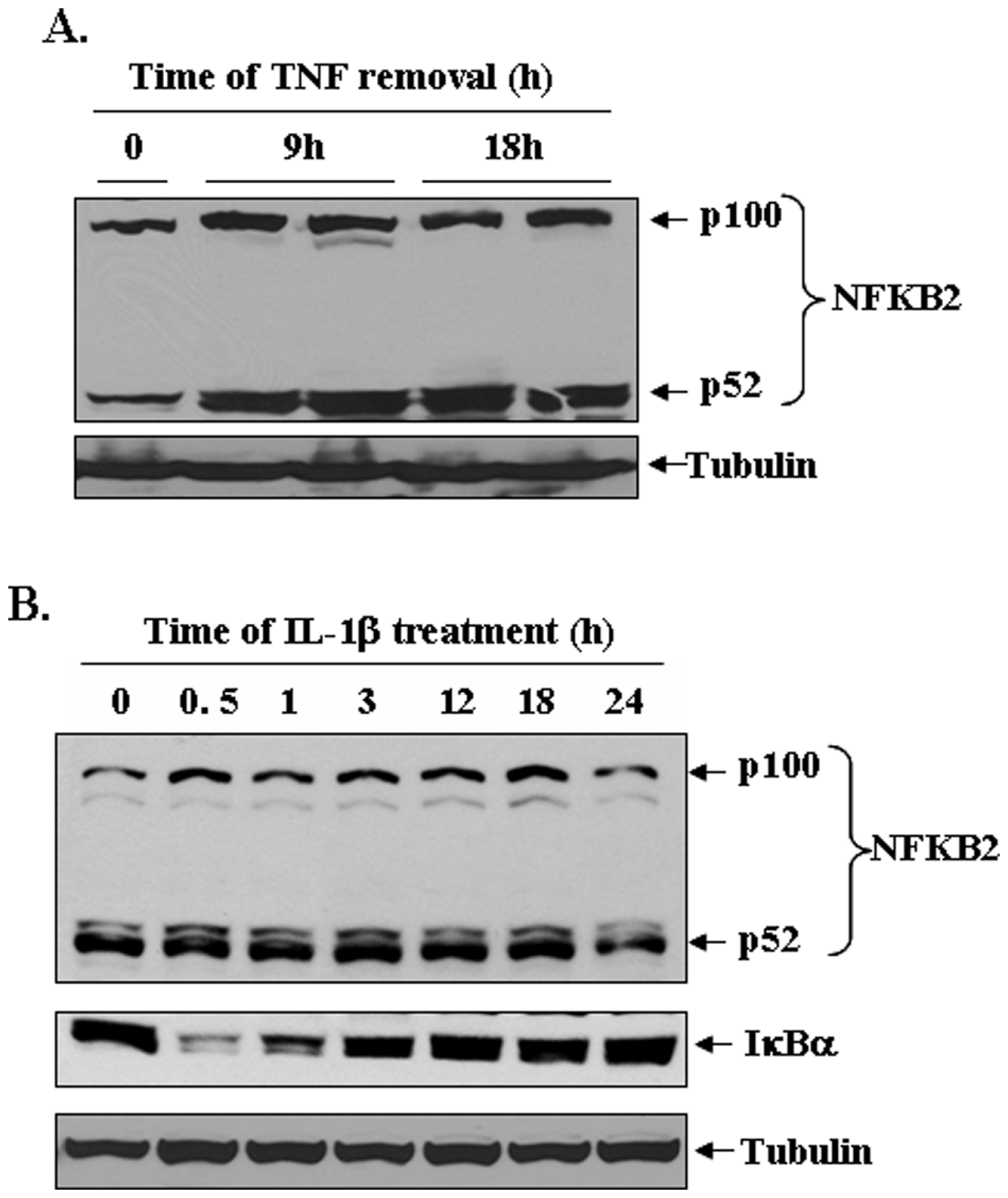
Role of TNF-α and IL-1β in the activation of alternative NF-κB signaling pathway. **A**). C2C12 myotubes were incubated with or without TNF-α (10 ng/ml) continuously for 18h or for only 9h followed by changing the medium with fresh differentiation medium without TNF-α and incubation for additional 9h. The cell lysate made were analyzed by Western blotting for p100/p52 protein. A representative immunoblot presented here demonstrate that removal of TNF-α after 9h did not affect the TNF-induced expression/processing of p100/p52 proteins. **B**). C2C12 myoblasts were treated with IL-1β (10 ng/ml) for indicated time periods and the levels of p100/p52 and IκBα protein were measured by Western blot. Data presented here show that IL-1β induced the degradation of IκBα protein (after 30 min and 1h) but did not affect the levels of either p100 or p52 proteins. TNF-α or IL-1β did not affect the levels of an unrelated protein tubulin in C2C12 cultures.

Although TNF-α was found to stimulate alternative NF-kB pathway, it was not clear whether TNF-α by itself is sufficient or other cytokines, chemokines, and growth factors produced in culture supernatants are responsible for the activation of alternative NF-κB pathway in TNF-treated myotubes. To address this issue, C2C12 myotubes were initially incubated with TNF-α for 9h, the medium of the cells was replaced with fresh differentiation medium (without TNF-α), and the cultures were incubated for additional 9h. In a parallel culture, C2C12 myotubes were continuously treated with TNF-α for 18h. At the end of the incubation period, the cells were collected and Western blot was performed using p100/p52 antibody. As shown in **Figure 7A**, TNF-α increased the levels of p100 and p52 protein in both the cultures (i.e. those incubated with TNF-α for 9h or 18h). Furthermore, we found that IL-1β (another inflammatory cytokine) activated classical NF-κB pathway evident by degradation of IκBα protein (at 30 min and 1h) but did not affect the protein levels of p100/p52 in C2C12 cultures (**Figure 7B**). Collectively, these results suggest that TNF-α alone may be sufficient to activate both classical and alternative pathways in C2C12 myotubes.

### Effect of TNF-α on various canonical pathways in C2C12 myotubes

**Figure 8:**
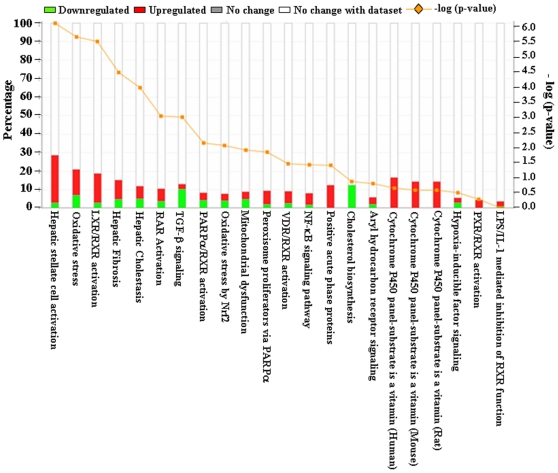
Differentially expressed genes associated with canonical pathway in the Ingenuity Pathway Analysis (IPA). Top canonical pathways affected by TNF-α treatment were identified by using IPA analysis. Here the differentially regulated genes with p-value ≤0.05 and fold change ≥1.2 were considered for IPA analysis. Bars represent −log p-value and percentage of genes present in the data set compared to the total number of genes present in each selected pathway in IPA data base. The yellow line represents −log p-value of affected genes to the total number of genes in a pathway. Each bar was represented in two different colors in which red correspond to the up-regulated genes and green corresponds to down-regulated genes. The percentage of up-regulated and down-regulated genes in each selected canonical pathway can be measured in percentage scale given on y-axis (left side).

Although TNF-α was found to differentially regulate the expression of a large number of genes, it was not clear how the expression of these genes affect the activity of various cellular and molecular pathways in skeletal muscle cells. To understand the effects of TNF on various canonical pathways, we used Ingenuity Pathway Analysis (IPA) software. We first used a set of differentially regulated genes with fold values ≥1.5 and p-value of ≤0.05 from microarray analyses as an input for IPA software. However, this set of genes was not sufficient to generate pathways affected by TNF-α. We then reduced the stringency and used the set of genes with fold change (both up- and down-regulated genes) values ≥1.2 and p-value of ≤0.05 in the microarray experiment. We found that TNF-α affected the expression of genes involved in specific molecular pathways in myotubes (**Figure 8**). The major pathways affected by TNF-α in myotubes were those that regulate hepatic fibrosis, LXR/RXR activity, oxidative stress, mitochondrial dysfunction, and TGF-β and NF-κB signaling (**Figure 8**). Interestingly, this bioinformatics analysis of pathways using differentially regulated genes is consistent with the experimental evidence that skeletal muscle-wasting and other muscular disorders such as muscular dystrophy, involve the activation of many of these molecular pathways [6], [10], [11], [33], [34].

### TNF-α regulates distinct gene networks in myotubes

**Figure 9:**
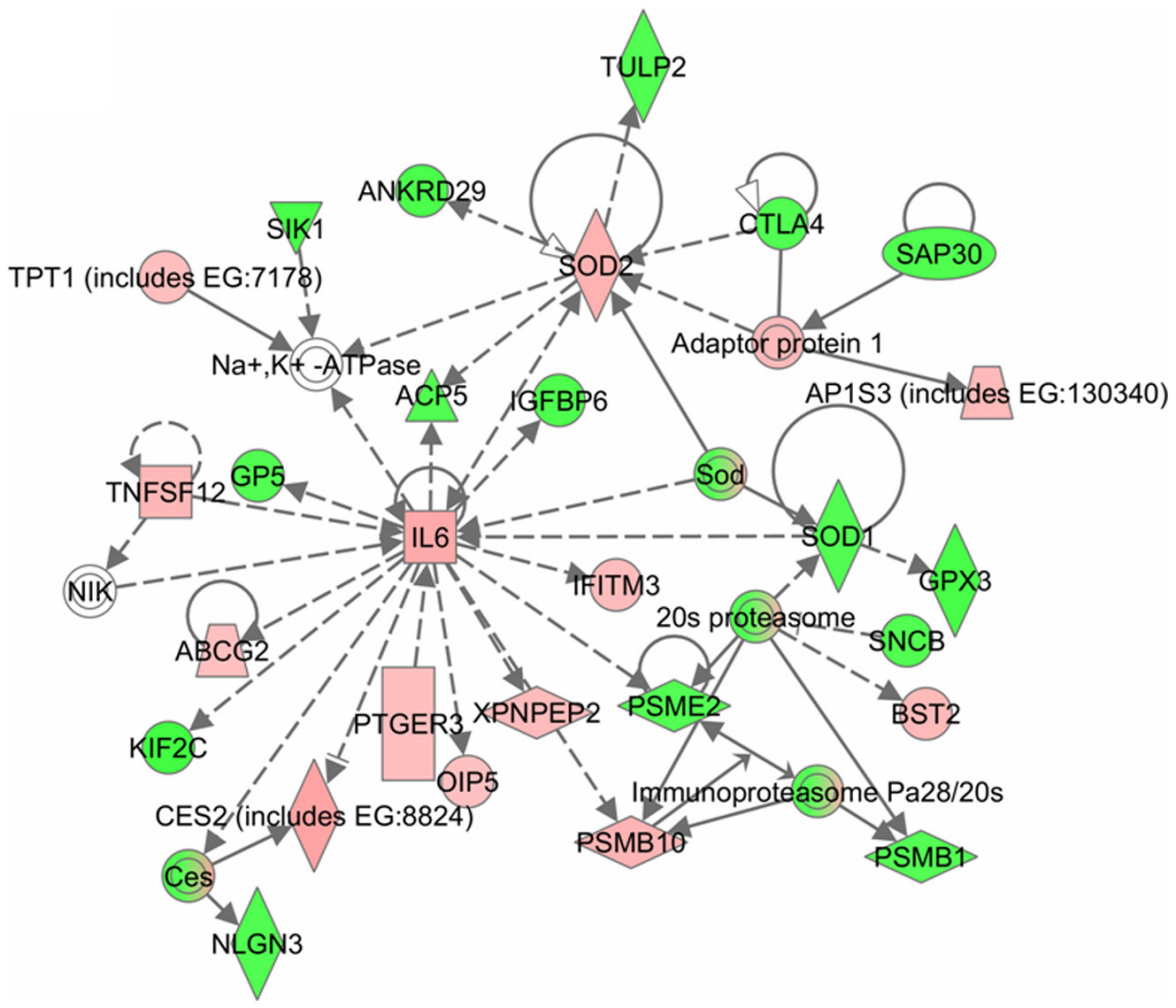
Network of genes involved in neurological disease and organismal injury in TNF-treated myotubes. The gene network presented here was adopted from Ingenuity pathway analysis tool with differentially regulated genes by TNF-α with p-values ≤0.05 and ≥1.2-fold. The solid lines connecting molecules here represent a direct relation and dotted lines an indirect relation. The genes shown in red are up-regulated whereas down-regulated genes are shown in green color.

**Figure 10:**
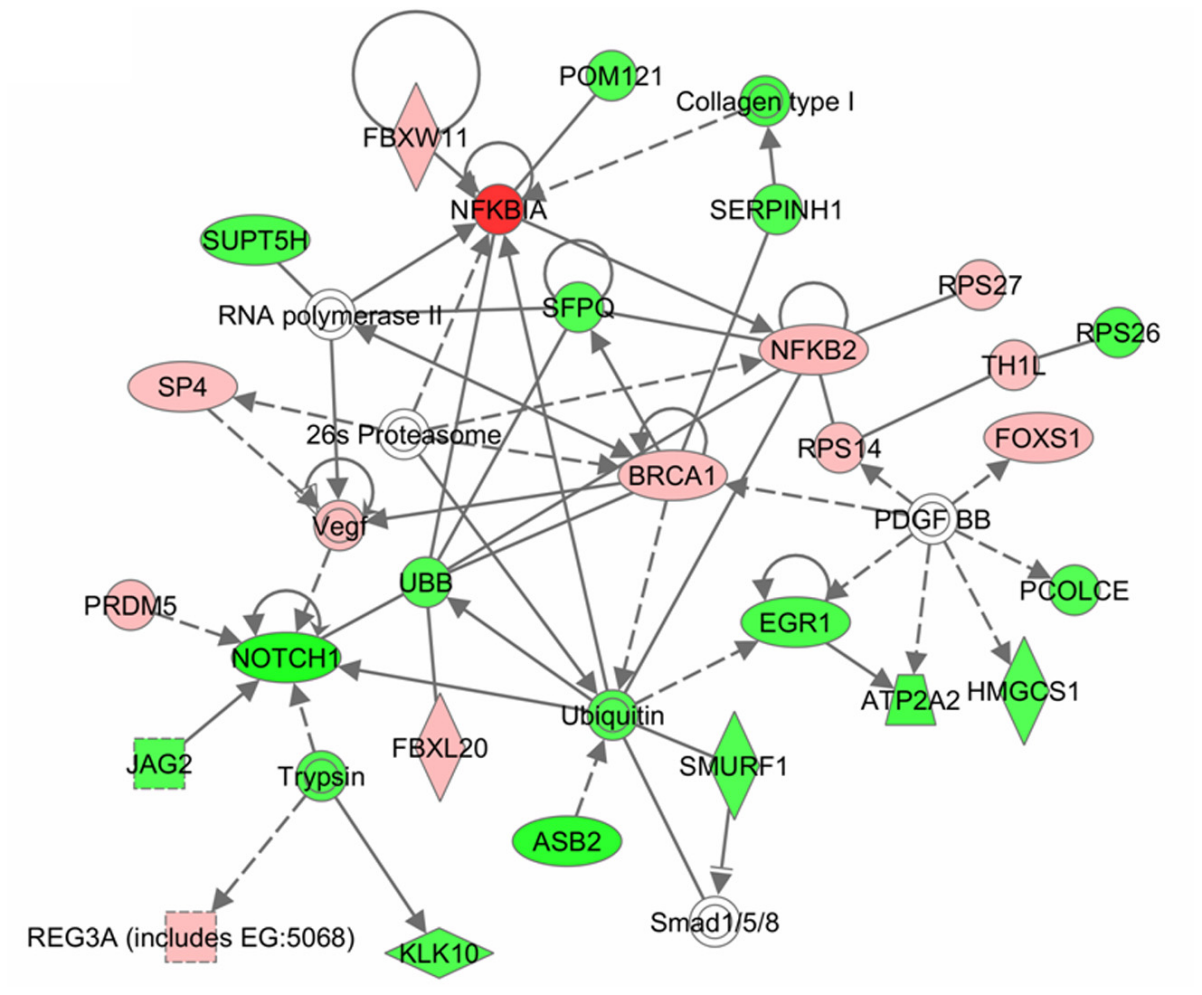
TNF-α regulates gene networks involved in nervous system development, cell cycle and cancer. Schematic representation of gene network obtained from Ingenuity pathway analysis tool with differentially regulated genes by TNF-α with p-values ≤0.05 and ≥1.2-fold. The solid lines connecting molecules here represent a direct relation and dotted lines are representation of an indirect relation. The genes shown in red color boxes are those which are up-regulated whereas down-regulated genes are shown in green color boxes.

In order to understand the interaction between different genes, we generated common networks using Ingenuity Pathway Analysis (IPA) software. The dataset of differentially expressed genes by TNF in C2C12 myotubes with selected stringency (p value ≤0.05 and fold ≥1.2) was uploaded into the IPA software tool. Networks of these genes were then algorithmically generated based on their connectivity. The graphical representation of the molecular relationships between genes developed by IPA is presented in **Figure 9** and **Figure 10**. Based on the input information, the genes that are down-regulated are shown in green and the up-regulated genes are shown in red (**Figure 9** and **Figure 10**). Cytokines, growth factors and oxidative stress enzymes such as TWEAK, IL-6, IGF binding protein (IGFBP), and SOD were found to be involved in the network related to drug metabolism, neurological disease, organismal injury and abnormalities (**Figure 9**). This network also showed that many of these genes are regulated by each other either directly or indirectly. Furthermore, the networks related to cell cycle, cancer and nervous system development and function showed that several genes such as Nfkb2, NFκBia, BRCA1, Vegf, Jag2, Notch1, EGR1, FoxS1 and collagen type I were regulated by TNF (**Figure 10**).

## Discussion

Although there is significant amount of literature suggesting the role of TNF-α in skeletal muscle wasting [3], [35], [36], [37], [38], the molecular mechanisms by which TNF-α induces muscle loss remain poorly understood. In this study, we have employed combination of microarray, bioinformatics tools, and biochemical techniques to identify potential mechanisms by which TNF-α might be regulating skeletal muscle mass.

### TNF-α induces the expression of several cytokines, chemokines, protein kinases, and transcriptional factors in cultured myotubes

The microarray analysis of TNF-treated C2C12 myotubes revealed that a total of 723 genes were significantly down regulated and 1,099 genes were significantly up regulated by TNF treatment with a p-value ≤0.05 and fold values of ≥1.2. Among the significantly up-regulated genes CXCL5 (8-fold), NFkB1 (1.6-fold), CCL2 (3.7-fold), CCL5 (3.6-fold), Vcam1 (3-fold), Taf2 (2.7-fold), Csf1 (2-fold), IL-6 (1.7-fold), Tnfaip3 or A20 (1.6-fold), MMP9 (1.5-fold), NFkB2 (1.4-fold), Traf5 (1.4-fold), TWEAK (1.4-fold), CXCR3 (1.37-fold), VEGF(1.28-fold), Brca1 (1.27-fold) and Creb5 (1.24-fold) are the genes having either direct or indirect role in many pathways induced by TNF-α. Infiltration of leukocytes is a characteristic feature of acute inflammatory condition which is found in many secondary myopathies. A study on large selection of alpha/beta-chemokines and their receptors in normal controls and in the inflammatory myopathies showed a general increase of specific chemokines and chemokine receptors [39]. An increase in expression levels of these chemokines and induction of neutrophil influx have been previously reported upon injection of TNF-α [40]. A recent study by Vieira et al [41] showed that intraperitoneal injection of KC/CXCL1 and LIX/CXCL5 induces dose and time-dependent neutrophil recruitment and TNF-α production. Consistent with these published reports, our microarray data show increased expression of many chemokine ligands and their receptors, especially, Cxcl5 which is up-regulated by 8-fold. The validation by QRT-PCR further confirmed the up-regulation of these chemokine networks by TNF-α (**Figure 2A**). These observations suggest that TNF-α induces the expression of chemokines and their receptors, leading to acute inflammation and myopathy.

Vascular cell adhesion molecule-1 (VCAM1), also known as CD106, is expressed in a number of tissues including skeletal muscle and its expression is increased in response to proinflammatory cytokines. It is one of the important target genes of NF-κB transcription factor [12]. The VCAM-1 protein mediates the adhesion of lymphocytes, monocytes, eosinophils, and basophils to different tissues. It also functions in leukocyte-endothelial cell signal transduction, and it may play a role in the development of atherosclerosis and rheumatoid arthritis. Recent studies of Do et al. [42] on microarray analysis of TNF treated human SGBS adipocytes also reveal the up-regulation of this inflammatory gene. Consistent with these studies, our microarray analysis of TNF-treated myotubes showed increased expression of VCAM1, which was further validated by QRT-PCR (**Figures 2A** **and** **4A**).

Interleukin IL-6 is an important cytokine which acts as both pro-inflammatory and anti-inflammatory molecule. This cytokine is also called as ‘myokine’ because it is also expressed by skeletal muscle and its levels are elevated during muscle contraction [43]. In one study, enhanced gene expression of IL-6 and decreased levels of insulin signaling pathway and Akt pathway was observed when enterocytes were treated with TNF-α [44]. Similar findings were observed in an independent study, where the infusion of angiotenisn II (AngII) increased circulating IL-6 in mice and triggered protein degradation via suppression of insulin signaling [45]. Our experiments demonstrate that TNF-α induces the expression of IL-6 in myotubes (**Figure 2A**) suggesting that TNF-α may also function through augmenting IL-6 expression to further accelerate inflammation and protein degradation in skeletal muscle.

### TNF suppresses the expression of genes involved in myogenesis, regeneration and ubiquitin proteasomal degradation

Our oligonucleotide microarray analyses of TNF-treated myotubes showed significant down-regulation of many important genes such as inhibitor of DNA binding 3 (−1.63-fold), Notch1 (−1.57-fold), FGF21 (−1.4-fold), procollagen type IV (−1.4-fold), phosphoglycerate mutase2 (−1.4-fold), protein kinase inhibitor, alpha (−1.38-fold), Myod1 (−1.3-fold), Smad6 (−1.3-fold), Mapk4 (−1.3-fold), Tgfβ3 (−.127-fold), FoxD2 (−.127-fold), adenylate cyclase 9 (−1.27-fold), Mef2c (−1.27-fold), Insulin-like 5 (−1.26-fold), Timp2 (−1.26-fold), Jagged 2 (−1.22-fold), Sod1 (−1.22-fold), Jun (−1.21-fold), and Tgfβ inducible early growth response (−.12-fold).

Recent studies have demonstrated that myogenic transcription factors such as serum response factor (SRF), MEF2c, and MyoD control the expression of myomiRs in skeletal and cardiac muscles [46]. Our microarray experiment showed that TNF-α inhibits the expression of MEF2c and MyoD transcription factors in cultured myotubes (**Table S1**). We have previously demonstrated that TWEAK reduces the levels of MyoD and myogenin in differentiating C2C12 cultures [47]. We have also shown that TWEAK down-regulates the expression levels of MEF2C in both microarray as well as QRT-PCR in C2C12 myotubes [28]. In correlation with these observations, we have also shown that TWEAK down-regulates the myomiRs regulated by MEF2c including miR-1, 133 and 206 [28]. The microarray analysis of TNF-treated myotubes in the present study showed a significant reduction in MEF2c and MyoD. The down-regulation of MyoD was further confirmed by QRT-PCR (**Figures 2A** **and** **4B**). From these observations, we can speculate that similar to TWEAK, TNF-α might also block myogenic differentiation by down-regulation of specific myogenic regulatory factors and thereby inhibiting the expression of various MyomiRs (miR-1, 133 and 206). These possibilities will be investigated in future studies.

We also observed down-regulation of an antioxidant enzyme, superoxide dismutase 1 (SOD1). SOD1 is a well-known player of the anti-oxidative defense. The direct evidence of SOD1 in muscular atrophy was recently reported by Muller et al. [48], where they showed a dramatic increase in mitochondrial reactive oxygen species (ROS) in three conditions of muscular atrophy in animals lacking *Sod1*. In another study, mutations in *Sod1*gene (SOD1^G93A^) selectively in skeletal muscle showed progressive muscle atrophy with concomitant reduction in muscle strength, alterations in contractile apparatus, and mitochondrial dysfunction [49]. In this study they also analyzed the molecular pathways associated with muscle atrophy by *Sod1* mutation and found that accumulation of oxidative stress initiated autophagy and thereby degradation of muscles. This suggests that skeletal muscle is the primary target of *Sod1* mutation-mediated toxicity whereby oxidative stress triggers muscle atrophy. The down-regulation of this important antioxidant in response to TNF-α treatment in our microarray data suggests a possible link between the regulation of *Sod1* and TNF-induced muscular atrophy.

Another important gene that was significantly down-regulated by TNF and is worth mentioning is Notch1. Notch-1 receptors are transmembrane proteins which are expressed in a broad range of tissues and function in diverse developmental and cell maturation processes [50], [51]. The intracellular regions of Notch receptors contain several functional motifs: ankyrin/CDC10 repeats, RAM, nuclear localization signals (NLS), PEST sequences, and a glutamine-rich domain [50]. Ligands such as Delta or Jagged/Serrate induce a second cleavage that requires presenilins at a site within the transmembrane region of Notch [52], [53] leading to release of the intracellular fragment of Notch, which binds to numerous nuclear and cytoplasmic proteins. Besides its role in regulation of the activity of other transcription factors, recent studies have shown that constitutively expressed Notch-1 functions as a novel IκB-like molecule and regulates NF-κB-mediated gene expression through a direct interaction with the NFkB1 (i.e. p50) subunit [54], [55]. This interaction prevents NF-κB from binding to NF-κB recognition sites in DNA to regulate NF-κB-dependent gene expression [54], [55]. Therefore, the reduced levels of Notch1 (**Figures 2B** **and** **5C**) may be responsible, at least in part, for sustained activation of NF-κB in skeletal muscle cells in response to TNF-α.

### TNF-α induces the activation of both canonical and alternative NF-κB signaling pathways in myotubes

Accumulating evidence strongly suggests that NF-κB is one the most important signaling pathways, the activation of which leads to skeletal muscle wastage [10]. NF-κB activation can occur through two parallel pathways. The canonical NF-κB signaling pathway involves the upstream activation of IκB kinase-β (IKKβ) and subsequent phosphorylation and degradation of IκB proteins [56], [57], [58]. In contrast, the activation of the alternative NF-κB pathway requires the upstream activation of NF-κB-inducing kinase (NIK or MAP3K14) and IKKα and the proteolytic processing of NFkB2 (p100 subunit) into p52 protein [57], [58]. Previous studies from our group and other laboratories have demonstrated that TWEAK increases the activation of both canonical and alternative NF-κB pathways [32], [59]. However, there has been no report on the activation of alternative NF-κB pathway by TNF-α in skeletal muscle. The microarray analysis in the present study showed that TNF-α increases the expression levels of both NFκB1 and NFκB2 in skeletal muscle cells (**Figures 2A** **,** **3A** **,** **4A** **,** **5A** **, and** **6B**). We also performed Western blots and EMSA and super-shift assays with radio-labeled NF-κB at different time points after the addition of TNF-α in cultured myotubes. These experiments demonstrated that TNF-α induces NF-κB activity via canonical pathway initially (**Figure 6B**). However a time-dependent increase in the components of alternative pathway was observed at later time points. These include the phosphorylation of IKKα, proteolytic degradation of p100 into p52 (**Figure 6B**), and presence of p52 and RelB subunits in NF-κB/DNA complex in super shift assays (**Figure 6C**). Since the activation of alternative pathway persisted even upon removal of TNF-α from culture medium after 9h (**Figure 7A**), these observations suggest that TNF-α alone might provide sufficient signal to activate alternative NF-κB pathway in skeletal muscle. It is also noteworthy that the activation of alternative NF-κB pathway in myotubes may be specific to TNF-α and TWEAK. This inference is supported by our findings that while IL-1β augmented the degradation of IκBα protein (an important event in the activation of classical pathway), it did not affect the levels of p100 and p52 proteins (**Figure 7B**). In future studies, it will be interesting to investigate which pathway mediates the loss of skeletal muscle mass in response to TNF-α.

### TNF-α affects multiple molecular pathways and gene networks in C2C12 myotubes

The Ingenuity pathway analysis (IPA) of the selected genes that are differentially regulated by TNF treatment in C2C12 myotubes showed that it affects the activation of multiple canonical pathways in skeletal muscle cells. The major pathways affected by TNF-α are those involved in initiation and manifestation of fibrosis, oxidative stress, and mitochondrial dysfunction (**Figure 8**). Interestingly, it has now become increasingly clear that skeletal muscle wasting in both chronic diseases and disuse conditions leads to the up-regulation of these pathways in skeletal muscle [10], [23], [60], [61] suggesting that TNF-α may be one of the important stimuli for their activation in catabolic conditions. Our analysis also showed that TNF-α affects the pathway involved in neurological disease and organismal injury (**Figure 9**) and nervous system development, cell cycle and cancer (**Figure 10**). Though TNF and TWEAK are two cytokines that share similar functions, it is of considerable importance that they affect distinct gene networks. Our previous studies showed the up-regulation of proteasomal pathway by TWEAK [28] but not by TNF-α. These studies further indicate that different inflammatory cytokines may regulate different set of molecular pathways in skeletal muscle and coordinated activation of these pathways may be responsible for the loss of skeletal muscle mass and myopathy in a particular disease state.

**Conclusions:** The data presented in this study suggest that TNF-α affects the expression of specific set of genes which are involved in the regulation of various molecular pathways/processes including extracellular matrix degradation, NF-κB signaling, Notch1 signaling, chemokine network, apoptosis, and muscle cell proliferation and differentiation. The study has also identified the activation of alternative NF-κB signaling pathway in C2C12 myotubes in response to TNF-α. The present study will provide strong basis for further delineating the molecular mechanisms of TNF-induced muscle wasting in different disease states.

## Materials and Methods

### Reagents

Dulbecco's modified Eagle's medium (DMEM), fetal bovine serum (FBS), Horse serum was purchased from Sigma Chemical Company (St. Louis, MO). Recombinant mouse TNF-α protein and antibodies against MMP-9 and TIMP2 were purchased from R&D Systems (Minneapolis, MN). Antibodies against IκBα, p50, p65, c-Jun, phospho-p52, phospho-IκB, phospho-IKKα and Notch1 were purchased from Santa Cruz Biotechnology (San Diego, CA). Tubulin, p52/100, RelB, IKKα, IKKβ and NFkB2 antibodies were obtained from Cell Signaling Technology (Beverly, MA). NF-κB consensus oligonucleotides and Dual-Luciferase assay kits were purchased from Promega (Madison, WI). Primers for PCR were synthesized by Integrated DNA Technologies (Coralville, IA) or Sigma-Genosys (Woodlands, TX). ^32^P-γ-ATP was obtained from MP Biomedicals (Solon, OH).

### Cell Culture

C2C12 myoblastic cell line was obtained from American Type Culture Collection (Rockville, MD). These cells were grown in Dulbecco's modified Eagle's Medium (DMEM) containing 20% fetal bovine serum. C2C12 myoblasts were differentiated into myotubes by incubation in differentiation medium (DM, 2% horse serum in DMEM) for 96h as described [47], [62]. Myotubes were maintained in DM and medium was changed every 48h.

### cDNA Microarray

Total RNA was isolated from control and TNF-treated C2C12 myotubes using the Agilent total RNA isolation kit (Agilent Technologies, Palo Alto, CA). Any contaminating DNA was removed using DNA-free™ kit from Ambion (Ambion, Austin, TX). The total RNA concentration was determined by NanoDrop spectrophotometer, and RNA quality was determined by 18S/28S ribosomal peak intensity on an Agilent Bioanalyzer. RNA samples from five wells per condition were separately subjected to microarray analyses.. Custom cDNA slides were spotted with Oligator “MEEBO” mouse genome set with 38,467 cDNA probes (Illumina, Inc., San Diego, CA), which allows interrogation of 25,000 genes. A Q-Array2 robot (Genetix) was used for spotting. The array includes positive controls, doped sequences, and random sequences to insure correct gene expression values were obtained from each array. A total of 250 ng RNA was used to synthesize double stranded cDNA using the Low RNA Input Fluorescent Linear Application Kit (Agilent). The microarray slides were scanned using a GSI Lumonics ScanArray 4200A Genepix scanner (Axon). The image intensities were analyzed using the ImaGene 5.6 software (Biodiscovery, Inc., El Segundo, CA). Expression analysis of microarray experiments was performed with GeneSpring 7.1 (Silicon Genetics, Palo Alto, CA) using the raw intensity data generated by the ImaGene software. Local background was subtracted from total signal intensities and was used as intensity measures. The data were normalized using per spot and per chip LOWESS normalization. Data analysis was performed using SAS (SAS Institute, Cary, NC), R and Q value software. The probe sets with absent calls across all samples were removed to reduce the multiple-testing problem. The expression levels were normalized to the chip median and log transformed. Two–way ANOVA tests were carried out to identify differentially expressed genes. For each probe set, the model 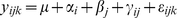 was fit, where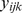 is the log-transformed expression level of the 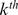 chip in the 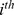 treatment and the 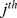 replicate. The variable 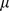 represents the grand mean expression, 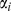 is the effect due to the treatment, 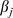 is the effect due to the replicate, 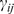 is the interaction effect between treatment and replicate, and 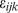 is an error term, which is assumed to be normally distributed with mean 0 and variance 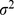. Q values computed using Q value software indicates the false detection rate for each probe set. Ratio comparison was performed by dividing expression levels in TNF-treated myotubes with the expression levels in untreated myotubes. Functional classification of select probe sets was performed at NIH DAVID server (http://apps1.niaid.nih.gov/david/upload.asp). Volcano plots were prepared using the R program. The complete raw and normalized microarray data have been submitted in MIAME compliant ArrayExpress (http://www.ebi.ac.uk/microarray-as/ae/) database with accession number E-MEXP-2592.

### Quantitative Real-Time-PCR (QRT-PCR)

The expression of the differentially regulated genes from the microarray data set was validated using QRT-PCR as previously described [47], [62]. Briefly, purified RNA (1 µg) from myotubes was used to synthesize first strand cDNA by reverse transcription system using Ambion's oligo-dT primer and Qiagen's Omniscript reverse transcriptase according to the manufacturer's instructions. The first strand cDNA reaction (0.5 µl) was subjected to real time PCR amplification using gene specific primers. The primers were designed using Vector NTI Xi software (Invitrogen).

Quantification of mRNA was done using the SYBR Green method on ABI Prism 7300 Sequence Detection System (Applied Biosystems, Foster City, CA). Approximately 25µl of reaction volume was used for the real time PCR assay that consisted of 2× (12.5µl) Brilliant SYBR Green QPCR Master Mix (Applied Biosystems), 400nM of primers (0.5 µl each from the stock), 11µl of water, and 0.5 µl of template. The thermal conditions consisted of an initial denaturation at 95°C for 10 minutes followed by 40 cycles of denaturation at 95°C for 15 sec, annealing and extension at 60°C for 1 minute, and a final step melting curve of 95°C for 15 sec, 60°C for 15 sec, and 95°C for 15 sec. All reactions were carried out in triplicate to reduce variation. The data was analyzed using SDS software version 2.0, and the results were exported to Microsoft Excel for further analysis. Data normalization was accomplished using the endogenous control β-actin and the normalized values were subjected to a 2^−ΔΔCt^ formula to calculate the fold change between the control and experimental groups. The formula and its derivations were obtained from the ABI Prism 7900 Sequence Detection System user guide.

### Pathways and Networks Analyses

Relative levels of gene expression were first computed with GeneSpring 7.1 to obtain data sets of differentially regulated genes based on cut-off values of 5% error rate (p<0.05, determined by t-test with Benjamini and Hochberg Multiple Testing Correction). These data sets included up and down regulated genes when C2C12 myotubes were treated with TNF. The second step of analysis consisted of identifying canonical pathways. Tab separated (txt) files containing Accession IDs and symbols derived from MEEBO genome set and the normalized expression ratios were then uploaded to Ingenuity Pathways Analysis. Ingenuity Pathways Analysis is a web-delivered bioinformatics tool (IPA 5.0, http://www.ingenuity.com) to identify pathways and functional networks. IPA knowledge database is generated from the peer-reviewed scientific publications that enables discovery. The Accession IDs and symbols in each data set were queried against all genes stored in the IPA knowledge database for pathway analysis. Canonical pathways analysis identified the pathways from IPA library of canonical pathways that were most significant to the data set. The significance of the association between the data set and the canonical pathways was measured in 2 ways: 1) A ratio of the number of genes from the data set that map to the pathway divided by the total number of genes that map to the canonical pathway is displayed. 2) Fisher's exact test was used to calculate a p-value determining the probability that the association between the genes in the data set and the canonical pathway is explained by chance alone.

### Western Blot

Western blotting was performed to measure the levels of different proteins using a standard protocol in our laboratory as described [32], [47], [62].The dilution of primary antibody was as follows: anti-IκB (1∶1000), anti-NFkB2 (1∶500), anti-MMP-9 (1∶2000), anti-p52/100 (1∶1000), anti-p50 (1∶1000), anti-RelB (1∶1000), anti-phospho IKKα (1∶500) anti-Notch1 (1∶1000), anti-TIMP2 (1∶500), and anti-tubulin (1∶3000). Immunoblots were quantified using ImageQuant TL software (GE Healthcare).

### Electrophoretic mobility shift assay (EMSA)

The activation of NF-κB transcription factors was measured by EMSA. A detailed procedure for the preparation of nuclear and cytoplasmic extracts and EMSA has been described previously [47].

### Statistical Analysis

Methods used for statistical analysis of the cDNA microarray has been described above. For all other studies, results were expressed as mean ± SD. The Student's *t* test was used to compare quantitative data populations with normal distributions and equal variance. A value of *P*<0.05 was considered statistically significant unless otherwise specified.

## Supporting Information

Table S1 (0.28 MB DOC)
