# Supplementary material for: Tumor Necrosis Factor-α Regulates Distinct Molecular Pathways and Gene Networks in Cultured Skeletal Muscle Cells
Source: PLoS One. 2010 Oct 12;5(10):e13262. doi: 10.1371/journal.pone.0013262 (PMC2953497; doi:10.1371/journal.pone.0013262)
Supplement: Table S1 — (0.28 MB DOC) [file pone.0013262.s001.doc]

| **Pathway**  **TABLE 1S**: Functional annotation of differentially expressed genes induced by TNF-α in C2C12 myotubes. | **Gene Name** | **p-value** | **Fold** | **Genbank** | **Gene Description** |
| --- | --- | --- | --- | --- | --- |
| Adipocytokine signaling pathway,  Apoptosis, B cell receptor signaling pathway,  MAPK signaling pathway, T cell receptor signaling pathway,  Toll-like receptor signaling pathway | Nfkb1 | 0.00059 | 1.60 | NM_008689 | nuclear factor of kappa light chain gene enhancer in B-cells 1, p105 |
|  | Nfkb2 | 0.00176 | 1.40 | NM_019408 | nuclear factor of kappa light polypeptide gene enhancer in B-cells 2, p49/p100 |
|  | Nfkbia | 1.69E-05 | 3.98 | NM_010907 | nuclear factor of kappa light chain gene enhancer in B-cells inhibitor, alpha |
| Aminosugars metabolism | Nans | 0.00396 | 1.23 | NM_053179 | N-acetylneuraminic acid synthase (sialic acid synthase) |
| Amyotrophic lateral sclerosis (ALS) and Neurodegenerative Disorders 01510 | Sod1 | 0.011 | -1.22 | NM_011434 | superoxide dismutase 1, soluble |
| Antigen processing and presentation, Cell adhesion molecules (CAMs),  Hematopoietic cell lineage, Type I diabetes mellitus | Psme2b | 0.000437 | 1.34 | NM_011191 | protease (prosome, macropain) 28 subunit, beta, b |
|  | H2-Ea | 0.0276 | 1.20 | NM_010381 | histocompatibility 2, class II antigen E alpha |
| Arachidonic acid metabolism, Glutathione metabolism 00480 | Gpx3 | 0.00295 | -1.29 | NM_008161 | glutathione peroxidase 3 |
| Basal transcription factors | Taf4a | 0.00125 | 1.49 | XM_130764 | TAF4A RNA polymerase II, TATA box binding protein (TBP)-associated factor |
| Calcium signaling pathway, Colorectal cancer, Cytokine-cytokine receptor interaction,  Focal adhesion, Gap junction, MAPK signaling pathway, Regulation of actin cytoskeleton | Pdgfrb | 0.0261 | 1.23 | NM_008809 | platelet derived growth factor receptor, beta polypeptide |
|  | Adcy9 | 0.00309 | -1.27 | NM_009624 | adenylate cyclase 9 |
|  | Calm4 | 0.00612 | -1.22 | NM_020036 | calmodulin 4 |
|  | Adora2a | 0.00904 | 1.33 | NM_009630 | adenosine A2a receptor |
|  | Htr6 | 0.00539 | -1.34 | NM_021358 | 5-hydroxytryptamine (serotonin) receptor 6 |
|  | Tacr3 | 0.00958 | 1.22 | NM_021382 | tachykinin receptor 3 |
| Cell adhesion molecules (CAMs), Leukocyte transendothelial migration | Cdh15 | 0.0018 | -1.32 | NM_007662 | cadherin 15 |
|  | Vcam1 | 0.0167 | 2.91 | NM_011693 | vascular cell adhesion molecule 1 |
| Cell Communication, ECM-receptor interaction,  Focal adhesion, Prion disease | Col1a2 | 0.00172 | -1.30 | NM_007743 | procollagen, type I, alpha 2 |
|  | Col4a2 | 0.00156 | -1.40 | NM_009932 | procollagen, type IV, alpha 2 |
|  | Lama2 | 0.001 | -1.26 | NM_008481 | laminin, alpha 2 |
|  | Lama1 | 0.019 | -1.23 | NM_008480 | laminin, alpha 1 |
| Cell cycle, Colorectal cancer, Cytokine-cytokine receptor interaction,  MAPK signaling pathway,TGF-beta signaling pathway | Cdkn1c | 0.000632 | -1.34 | NM_009876 | cyclin-dependent kinase inhibitor 1C (P57) |
|  | Tgfb3 | 6.98E-05 | -1.29 | NM_009368 | transforming growth factor, beta 3 |
| Cytokine-cytokine receptor interaction, Hematopoietic cell lineage,  Jak-STAT signaling pathway, Toll-like receptor signaling pathway | Ccl2 | 0.000105 | 3.77 | NM_011333 | chemokine (C-C motif) ligand 2 |
|  | Ccl24 | 0.0409 | 1.34 | NM_019577 | chemokine (C-C motif) ligand 24 |
|  | Ccl7 | 0.0141 | 1.45 | NM_013654 | chemokine (C-C motif) ligand 7 |
|  | Ccr7 | 0.0174 | -1.23 | NM_007719 | chemokine (C-C motif) receptor 7 |
|  | Cxcl5 | 7.54E-05 | 8.04 | NM_009141 | chemokine (C-X-C motif) ligand 5 |
|  | Cxcr3 | 0.00305 | 1.38 | NM_009910 | chemokine (C-X-C motif) receptor 3 |
|  | Il18r1 | 0.0403 | 1.21 | NM_008365 | interleukin 18 receptor 1 |
|  | Il8rb | 0.00121 | 1.21 | NM_009909 | interleukin 8 receptor, beta |
|  | Tnfsf12 | 0.029 | 1.39 | NM_011614 | tumor necrosis factor (ligand) superfamily, member 12 |
|  | Vegfc | 0.000288 | 1.28 | NM_009506 | vascular endothelial growth factor C |
|  | Csf1 | 0.000597 | 2.02 | NM_007778 | colony stimulating factor 1 (macrophage) |
|  | CSF-1 | 0.0185 | 1.29 | X05010 | Mouse mRNA for colony stimulating factor-1 CSF-1 (M-CSF). |
|  | Il6 | 0.00708 | 1.66 | NM_031168 | interleukin 6 |
|  | Ccl5 | 0.000286 | 3.59 | NM_013653 | chemokine (C-C motif) ligand 5 |
| Dorso-ventral axis formation, Notch signaling pathway | Notch1 | 0.00137 | -1.57 | NM_008714 | Notch gene homolog 1 (Drosophila) |
|  | Notch3 | 0.021 | -1.20 | NM_008716 | Notch gene homolog 3 (Drosophila) |
|  | Jag2 | 0.000906 | -1.22 | NM_010588 | jagged 2 |
| Focal adhesion | Cav1 | 0.0339 | -1.26 | NM_007616 | caveolin, caveolae protein 1 |
| Fructose and mannose metabolism | Pgm1 | 0.00239 | 1.21 | NM_025700 | phosphoglucomutase 1 |
| Gap junction | Tuba1 | 0.0033 | 1.20 | NM_011653 | tubulin, alpha 1 |
| Glutamate metabolism, Nitrogen metabolism, Peptidoglycan biosynthesis | Glul | 0.0193 | -1.22 | NM_008131 | glutamate-ammonia ligase (glutamine synthase) |
| Glycolysis / Gluconeogenesis | Pgam2 | 0.00351 | -1.39 | NM_018870 | phosphoglycerate mutase 2 |
| Hedgehog signaling pathway, Wnt signaling pathway | Wnt10a | 0.0058 | 1.30 | NM_009518 | wingless related MMTV integration site 10a |
|  | Wnt3 | 0.00153 | -1.25 | NM_009521 | wingless-related MMTV integration site 3 |
| Inositol phosphate metabolism | Isyna1 | 0.0283 | 1.29 | NM_023627 | myo-inositol 1-phosphate synthase A1 |
| Jak-STAT signaling pathway | Stat5a | 0.00379 | 1.22 | NM_011488 | signal transducer and activator of transcription 5A |
| B cell receptor signaling pathway, Colorectal cancer, Focal adhesion,  GnRH signaling pathway, MAPK signaling pathway, T cell receptor signaling pathway,  Toll-like receptor signaling pathway, Wnt signaling pathway | Jun | 0.0334 | -1.22 | NM_010591 | Jun oncogene |
| Extracellular matrix | Timp1 | 7.73E-05 | 1.61 | NM_011593 | tissue inhibitor of metalloproteinase 1 |
|  | Timp2 | 0.0278 | -1.26 | NM_011594 | tissue inhibitor of metalloproteinase 2 |
|  | Mmp9 | 0.00572 | 1.50 | NM_013599 | matrix metalloproteinase 9 |
| MAPK signaling pathway | Cacng6 | 0.0104 | 1.24 | NM_133183 | calcium channel, voltage-dependent, gamma subunit 6 |
|  | Mef2c | 0.019 | -1.27 | NM_025282 | Mef2c protein. |
|  | Fgf21 | 0.00342 | -1.42 | NM_020013 | fibroblast growth factor 21 |
| Neuroactive ligand-receptor interaction | Gabrb2 | 0.0345 | 1.22 | NM_008070 | gamma-aminobutyric acid (GABA-A) receptor, subunit beta 2 |
|  | Gabrr1 | 0.0117 | 1.23 | NM_008075 | gamma-aminobutyric acid (GABA-C) receptor, subunit rho 1 |
|  | Galr2 | 0.00392 | -1.28 | NM_010254 | galanin receptor 2 |
|  | Glp1r | 0.00454 | -1.26 | NM_021332 | glucagon-like peptide 1 receptor |
|  | Grid1 | 0.0292 | 1.22 | NM_008166 | glutamate receptor, ionotropic, delta 1 |
|  | Grin3b | 0.0194 | 1.25 | NM_130455 | glutamate receptor, ionotropic, NMDA3B |
|  | Mc4r | 0.00378 | 1.22 | NM_016977 | melanocortin 4 receptor |
|  | Npy1r | 0.0313 | 1.24 | NM_010934 | neuropeptide Y receptor Y1 |
| Proteasome | Psmb1 | 0.0106 | -1.21 | NM_011185 | proteasome (prosome, macropain) subunit, beta type 1 |
| Regulation of autophagy | Gabarap | 0.000901 | 1.20 | NM_019749 | gamma-aminobutyric acid receptor associated protein |
| Breast cancer induced | Brca1 | 0.00954 | 1.27 | NM_009764 | breast cancer 1 |
| Transcriptional factors | Creb5 | 0.0185 | 1.24 | NM_172728 | cAMP responsive element binding protein 5 |
|  | Egr1 | 0.0075 | -1.28 | NM_007913 | early growth response 1 |
|  | Fkhl18 | 0.0159 | 1.33 | NM_010226 | forkhead-like 18 (Drosophila) |
|  | Foxd2 | 0.00074 | -1.28 | NM_008593 | forkhead box D2 |
|  | Foxd3 | 0.00277 | 1.22 | NM_010425 | forkhead box D3 |
|  | Tcf4 | 2.03E-05 | -1.58 | NM_013685 | transcription factor 4 |
|  | Foxo6 | 0.00739 | -1.22 | NM_194060 | forkhead box O6 |
| Cell cycle | H2afv | 0.00206 | -1.49 | XM_126043 | H2A histone family, member V |
|  | H2afz | 0.00637 | -1.25 | NM_016750 | H2A histone family, member Z |
|  | Hdac10 | 0.0107 | -1.21 | NM_199198 | histone deacetylase 10 |
|  | Hdac7a | 0.0155 | 1.23 | NM_019572 | histone deacetylase 7A |
|  | Hist1h2bh | 0.0383 | -1.26 | NM_178197 | histone 1, H2bh |
|  | Hist1h2bl | 0.0125 | -1.23 | NM_178199 | histone 1, H2bl |
|  | Hist1h2bp | 0.00117 | -1.24 | NM_178202 | histone 1, H2bp |
|  | Hist2h2be | 0.0139 | -1.25 | NM_178214 | histone 2, H2be |
|  | Hist2h3c2 | 0.0063 | -1.25 | NM_054045 | histone 2, H3c2 |
| IGF-signaling | Igfbp6 | 0.00369 | -1.25 | NM_008344 | insulin-like growth factor binding protein 6 |
|  | Igfbp7 | 0.000234 | 1.57 | NM_008048 | insulin-like growth factor binding protein 7 |
|  | Igfbpl1 | 0.00189 | -1.20 | NM_018741 | insulin-like growth factor binding protein-like 1 |
|  | Il1f10 | 0.0311 | 1.21 | NM_153077 | interleukin 1 family, member 7 |
|  | Inha | 0.0212 | -1.24 | NM_010564 | inhibin alpha |
|  | Insl5 | 0.00581 | -1.27 | NM_011831 | insulin-like 5 |
|  | Itpk1 | 0.0313 | 1.21 | NM_172584 | inositol 1,3,4-triphosphate 5/6 kinase |
|  | Lox | 0.0187 | 1.21 | NM_010728 | lysyl oxidase |
|  | Mapk4 | 0.014 | -1.29 | NM_172632 | mitogen-activated protein kinase 4 |
|  | Mbnl3 | 0.017 | 1.23 | NM_134163 | muscleblind-like 3 (Drosophila) |
|  | Mcpt6 | 0.00913 | 1.23 | NM_010781 | mast cell protease 6 |
|  | Mill1 | 0.0283 | 1.27 | NM_153749 | MHC I - like leukocyte 1 |
|  | Mybpc3 | 0.038 | 1.21 | NM_008653 | myosin binding protein C, cardiac |
|  | Myh11 | 0.00504 | 1.24 | NM_013607 | myosin heavy chain 11, smooth muscle |
|  | Myo18a | 0.0131 | -1.23 | NM_011586 | myosin XVIIIa |
|  | Myod1 | 0.0198 | -1.32 | NM_010866 | myogenic differentiation 1 |
|  | Neurog2 | 0.0375 | 1.22 | NM_009718 | neurogenin 2 |
|  | Nfrkb | 0.000189 | -1.27 | NM_172766 | nuclear factor related to kappa B binding protein |
|  | Ngfrap1 | 0.000899 | -1.30 | NM_009750 | nerve growth factor receptor (TNFRSF16) associated protein 1 |
|  | Nid2 | 0.000535 | -1.32 | NM_008695 | nidogen 2 |
|  | Ninj1 | 0.0152 | 1.26 | NM_013610 | ninjurin 1 |
|  | Nmyc1 | 0.00404 | 2.01 | NM_008709 | neuroblastoma myc-related oncogene 1 |
|  | Nrap | 0.00118 | -1.26 | NM_008733 | nebulin-related anchoring protein |
|  | Obscn | 0.0127 | -1.32 | NM_001003914 | obscurin, cytoskeletal calmodulin and titin-interacting RhoGEF |
|  | Pex1 | 7.44E-05 | 1.37 |  | peroxisome biogenesis factor 1 |
|  | Pkia | 0.000608 | -1.38 | NM_008862 | protein kinase inhibitor, alpha |
|  | Psmb10 | 0.0111 | 1.46 | NM_013640 | proteasome (prosome, macropain) subunit, beta type 10 |
|  | Rab3a | 0.000624 | -1.28 | NM_009001 | RAB3A, member RAS oncogene family |
|  | Rnf7 | 0.000658 | -1.32 | XM_135065 | ring finger protein 7 |
|  | Sart2 | 0.00347 | 1.35 | NM_172508 | squamous cell carcinoma antigen recognized by T cells 2 |
|  | Snapc5 | 0.0037 | 1.29 | NM_183316 | small nuclear RNA activating complex, polypeptide 5 |
|  | Taf2 | 0.00289 | 2.70 |  | TAF2 RNA polymerase II, TATA box binding protein (TBP)-associated factor, 150kDa |
| TNF-alpha signaling | Tnfaip3 | 0.000275 | 1.57 | NM_009397 | tumor necrosis factor, alpha-induced protein 3 |
|  | Tnfrsf23 | 0.00245 | -1.25 | NM_024290 | tumor necrosis factor receptor superfamily, member 23 |
|  | Traf5 | 0.000748 | 1.40 | NM_011633 | Tnf receptor-associated factor 5 |
|  | Trp53inp2 | 0.0165 | 1.37 | NM_178111 | tumor protein p53 inducible nuclear protein 2 |
|  | Ube2e2 | 0.00284 | 1.25 | NM_144839 | ubiquitin-conjugating enzyme E2E 2 (UBC4/5 homolog, yeast) |
|  | Usmg4 | 2.71E-05 | -1.33 | NM_031401 | upregulated during skeletal muscle growth 4 |
|  | Usp27x | 0.0245 | 1.23 | NM_019461 | ubiquitin specific protease 27, X chromosome |
|  | Usp40 | 0.00255 | -1.22 | XM_129956 | ubiquitin specific protease 40 |
|  | Usp49 | 0.022 | -1.29 | NM_020048 | ubiquitin specific protease 49 |
|  | Wisp2 | 0.018 | -1.32 | NM_016873 | WNT1 inducible signaling pathway protein 2 |
| TGF-beta signaling pathway | Tieg1 | 0.0136 | 1.22 | NM_013692 | TGFB inducible early growth response 1 |
|  | Tieg3 | 0.00485 | -1.21 | NM_178357 | TGFB inducible early growth response 3 |
|  | Idb3 | 0.000245 | -1.63 | NM_008321 | inhibitor of DNA binding 3 |
|  | Smad6 | 0.000131 | -1.31 | NM_008542 | MAD homolog 6 (Drosophila) |
|  | Smurf1 | 0.000129 | -1.23 | NM_029438 | SMAD specific E3 ubiquitin protein ligase 1 |
